# Crystal structure, hydrogen bonding and Hirshfeld surface analysis of 2-amino-4-meth­oxy-6-methyl­pyrimidinium 4-chloro­benzoate

**DOI:** 10.1107/S2056989018005583

**Published:** 2018-04-12

**Authors:** Muthaiah Jeevaraj, Palaniyappan Sivajeyanthi, Bellarmin Edison, Kaliyaperumal Thanigaimani, Kasthuri Balasubramani

**Affiliations:** a Department of Chemistry, Government Arts College (Autonomous), Thanthonimalai, Karur 639 005, Tamil Nadu, India; b Department of Chemistry, Government Arts College, Tiruchirappalli 620 022, Tamil Nadu, India

**Keywords:** crystal structure, pseudo­tetra­meric, pyrimidine

## Abstract

In the crystal structure of the title salt, C_6_H_10_N_3_O^+^·C_7_H_4_ClO_2_^−^, the dihedral angle between the pyrimidine ring of the 2-amino-4-meth­oxy-6-methyl­pyrimidine cation and the the benzene ring of the 2-chloro­benzoate anion is 2.2 (1)°. In the anion, the benzene ring forms a dihedral angle of 8.5 (2)° with the carboxyl group. The pyrimidine N atom of the cation is protonated and the meth­oxy substituent is essentially coplanar with the parent ring. The protonated N atom and the N atom of the 2-amino group are hydrogen bonded to the 4-chloro­benzoate anion through a pair of N—H⋯O_carbox­yl_ hydrogen bonds, forming an *R* ^2^_2_(8) ring motif linked through a centrosymmetric *R* ^2^_4_(8) ring motif, resulting in a pseudo­tetra­meric *DDAA* array. These units are linked through inter­molecular meth­oxy C—H⋯Cl hydrogen bonds into ribbon-like chains extending along the *c*-axis direction. The crystal structure also features π–π stacking inter­actions between the rings in the cation and anion [minimum ring centroid separation = 3.7707 (12) Å].

## Abstract 2

In the crystal structure of the title compound, C_6_H_10_N_3_O^+^·C_7_H_4_Cl_O_2^−^, the pyrimidine N atom of the cation is hydrogen-bonded to the 4-chloro­benzoate anion through a pair of N—H⋯O_carbox­yl_ hydrogen bonds, forming an 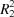
(8) ring motif which is linked through centrosymmetric 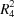
(8) ring motifs, forming a pseudo­tetra­meric *DDAA* array.

## Chemical context   

Pyrimidine and amino­pyrimidine derivatives are biologically important compounds and they occur in nature as components of nucleic acids such as cytosine, uracil and thymine. Pyrimidine derivatives are also important mol­ecules in biology and have many applications in the areas of pesticides and pharmaceutical agents (Condon *et al.*, 1993 ▸). For example, imazosulfuron, ethirmol and mepanipyrim have been commercialized as agrochemicals (Maeno *et al.*, 1990 ▸). Pyrimidine derivatives have also been developed as anti­viral agents, such as AZT, which is the most widely used anti-AIDS drug (Gilchrist, 1997 ▸). In order to study the hydrogen-bonding inter­actions, the title compound, the 2-amino-4-meth­oxy-6-methyl­pyrimidinium salt of 4-chloro­benzoate, C_6_H_10_N_3_O^+^·C_7_H_4_ClO_2_^−^, was synthesized and its structure, hydrogen-bonding and Hirshfeld surface analysis are reported herein.
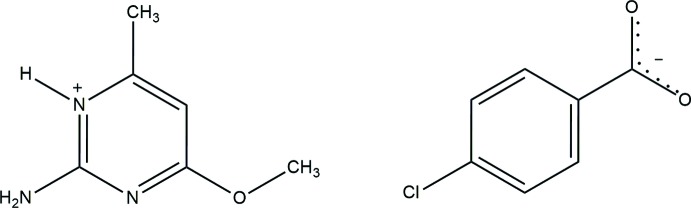


## Structural commentary   

**Figure 1:**
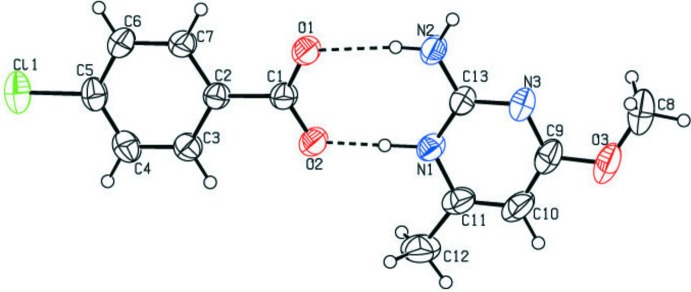
The asymmetric unit of the the title compound with atom labels, showing non-hydrogen atoms as 30% probability displacement ellipsoids. Inter-species hydrogen bonds are shown as dashed lines.

The asymmetric unit of the title compound contains a 2-amino-4-meth­oxy-6-methyl­pyrimidinium cation and a 4-chloro­benzoate anion (Fig. 1 ▸), which are essentially coplanar, with a dihedral angle between the ring systems of the two species of 2.2 (1)°. In the cation, one of the pyrimidine nitro­gen atoms (N1) is protonated and this is reflected in an increase in bond angle at N1 [C11—N1—C13 = 120.53 (17)°], when compared with that at the unprotonated atom (N3) [C9—N3—C13 = 116.32 (18)°] and the corresponding angle of 116.01 (18)° in neutral 2-amino-4-meth­oxy-6-methyl­pyrimidine (Glidewell *et al.*, 2003 ▸). The meth­oxy substituent group at C9 of the cation is essentially coplanar with the ring, the N3—C9—O3—C8 torsion angle being −2.9 (3)°. The bond lengths and angles are normal for the carboxyl­ate group of a 4-chloro­benzoate anion, and the benzene ring forms a dihedral angle of 8.5 (2)° with the carboxyl group.

## Supra­molecular features   

**Figure 2:**
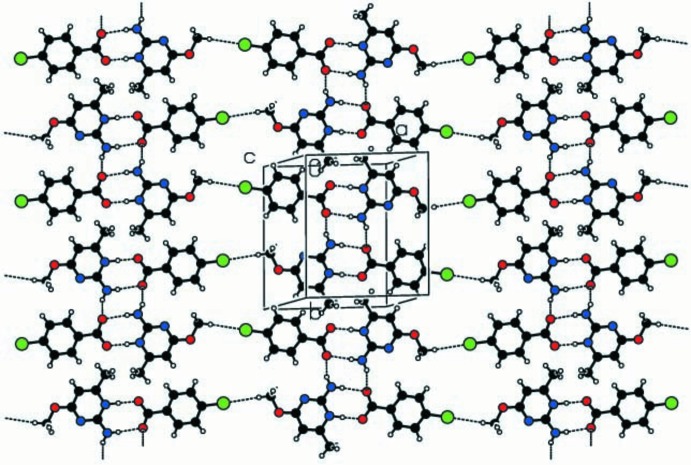
Hydrogen bonding in the structure of the title compound showing the 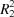
(8) and centrosymmetric 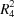
(8) ring motifs and C—H⋯Cl extensions. Dashed lines indicate the hydrogen bonds.

**Figure 3:**
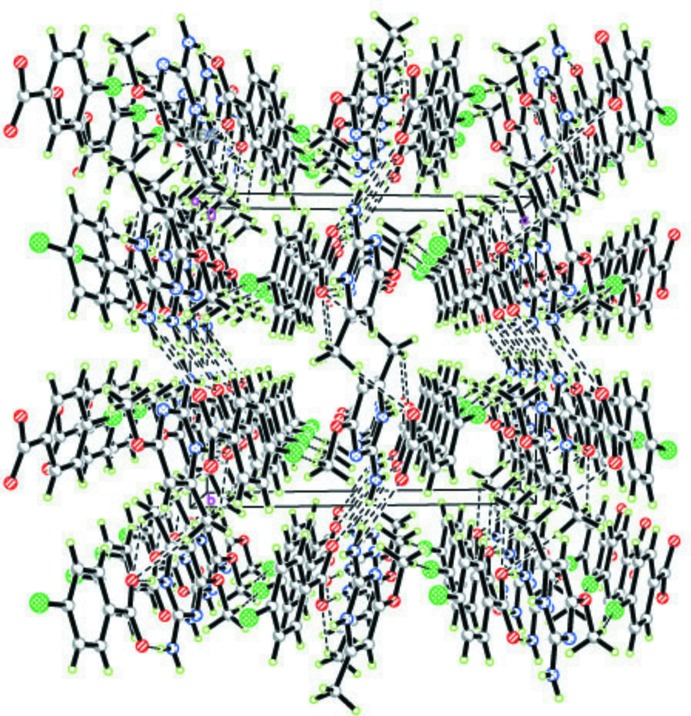
The overall view of the packing and stacking inter­actions in the title compound.

In the crystal, the protonated nitro­gen atom (N1) and the amino nitro­gen atom (N2) of the cation inter­act with the carboxyl oxygen atoms O2 and O1, respectively, of the anion through N—H⋯O hydrogen bonds (Table 1 ▸), forming an eight-membered 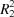
(8) ring motif. This is extended into a *DDAA* array (where *D* represents a hydrogen-bond donor and *A* represents a hydrogen-bond acceptor) by N2—H1*N*⋯O1^i^ hydrogen bonds in a centrosymmetric 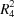
(8) association [symmetry code: (i) −*x* + 1, −*y* + 2, −*z* + 1], the corresponding graph-set notations for the hetero­tetra­mer being 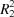
(8), 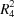
(8), 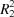
(8). The hetero­tetra­meric units are linked through meth­oxy C8—H8*A*⋯Cl^ii^ hydrogen bonds, forming one-dimensional ribbon-like structures (Fig. 2 ▸) [symmetry code: (ii) *x* + 2, −*y* + 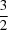, *z* + 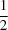]. Only very weak methyl C12—H⋯O2 inter­actions [C⋯O = 3.442 (3) Å; H⋯O2 = 2.76 Å] exist between ribbons. The crystal structure also features π–π stacking inter­actions between the aromatic pyrimidine ring of the cation (Fig. 3 ▸) and the benzene ring of the anion, with minimum centroid–centroid and perpendicular inter­planar distances of 3.7780 (12) and 3.7075 (8) Å, respectively, and a slip angle of 19.44° (Hunter *et al.*, 1994 ▸).

## Hirshfeld surface analysis   

**Figure 4:**
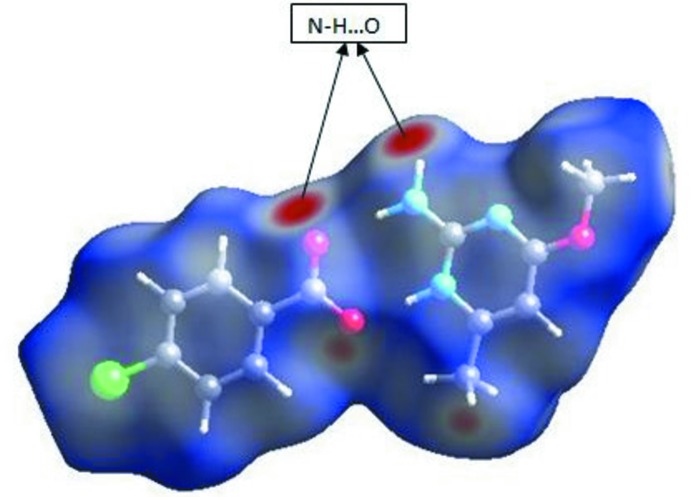
The three-dimensional *d*_norm_ surface of the title compound.

**Figure 5:**
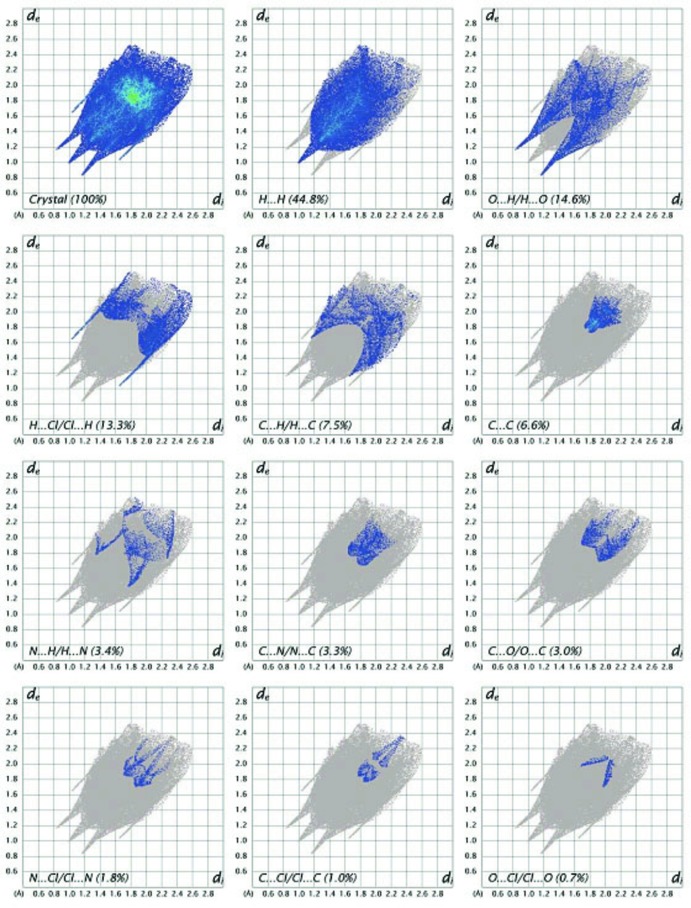
Two-dimensional fingerprint plots with the relative contributions to the Hirshfeld surface.

Three-dimensional (3D) *d*_norm_ surface analyis is a useful tool for analysing and visualizing the inter­molecular inter­actions. *d*_norm_ takes negative or positive values depending on whether the inter­molecular contact is shorter or longer, respectively, than the van der Waals radii (Spackman & Jayatilaka, 2009 ▸; McKinnon *et al.*, 2007 ▸). The 3D *d*_norm_ surface of the title compound was shown in Fig. 4 ▸. The red points represent closer contacts and negative *d*_norm_ values on the surface corres­ponding to the N—H⋯O interactions, while C—H⋯O inter­actions are light red in colour. Two-dimensional fingerprint plots from the Hirshfeld surface analysis are shown in Fig. 5 ▸, revealing the inter­molecular contacts and their percentage distributions on the Hirshfeld surface. H⋯H inter­actions (44.8%) are present as a major contributor while O⋯H/H⋯O (14.6%), H⋯Cl/Cl⋯H (13.3%), C⋯H/H⋯C (7.5%), C⋯C (6.6%), N⋯H/H⋯N (3.4%), C⋯N/N⋯C (3.3%), Cl⋯N/N⋯Cl (1.8%), C⋯Cl/Cl⋯C (1.0%) and Cl⋯O/O⋯Cl (0.7%) contacts also make significant contributions to the Hirshfeld surface. Two ‘wingtips’ in the fingerprint plot are related to H⋯O and O⋯H inter­actions and are shown in Fig. 5 ▸.

## Database survey   

A search of the Cambridge Structural Database (Version 5.37, update February 2017; Groom *et al.*, 2016 ▸) for 2-amino-4-meth­oxy-6-methyl­pyrimidine yielded only seven structures of proton-transfer salts with carb­oxy­lic acids: VAQSOW [with 3-(*N,N*-di­methyl­amino)­benzoic acid]; VAQSUC [with methyl­ene hydrogen succinic acid (a monohydrate)]; VAQSEM (with 3-nitro­benzoic acid); VAQSIQ (with benzoic acid); VAQRUB (with 2-fluoro­benzoic acid) and VAQSAI (with 3-chloro­benzoic acid) (all from Aakeröy *et al.*, 2003 ▸) and NUQTOJ (with picric acid; Jasinski *et al.*, 2010 ▸).

## Synthesis and crystallization   

The title compound was synthesized by the reaction of a 1:1 stoichiometric mixture of 2-amino-4-meth­oxy-6-methyl­pyrimidine [0.139 mg (Aldrich)] and 4-chloro­benzoic acid [0.156 mg (Merck)] in 20 ml of a hot methano­lic solution. After warming for a few minutes over a water bath, the solution was cooled and kept at room temperature. Within a few days, colourless block-shaped crystals suitable for the X-ray analysis were obtained (yield: 65%).

## refinement   

Crystal data, data collection and structure refinement details are summarized in Table 2 ▸. N-bound pyrimidinium H atoms were located in a difference-Fourier map and refined freely [N—H = 1.03 (3) Å]. The remaining H atoms were positioned geometrically and refined using a riding model with (N—H = 0.86 Å and C—H = 0.93 or 0.96 Å) and *U*_iso_(H) = 1.2 *U*_eq_(C,N) or 1.5*U*_eq_(methyl C). A rotating-group model was used for the methyl groups.

## Supplementary Material

Crystal structure: contains datablock(s) global, I, 81R. DOI: 10.1107/S2056989018005583/zs2399sup1.cif

Structure factors: contains datablock(s) I. DOI: 10.1107/S2056989018005583/zs2399Isup2.hkl

Supporting information file. DOI: 10.1107/S2056989018005583/zs2399Isup3.cml

CCDC reference: 1835970

Additional supporting information:  crystallographic information; 3D view; checkCIF report

## Figures and Tables

**Table 1:** Hydrogen-bond geometry (Å, °)

*D*—H⋯*A*	*D*—H	H⋯*A*	*D*⋯*A*	*D*—H⋯*A*
N1—H1*N*1⋯O2	1.04 (3)	1.60 (3)	2.636 (3)	176 (2)
N2—H1*N*⋯O1^i^	0.86	2.12	2.846 (2)	142
N2—H2*N*⋯O1	0.86	1.97	2.824 (3)	169
C8—H8*A*⋯Cl1^ii^	0.96	2.82	3.770 (3)	171

Symmetry codes: (i) 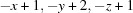; (ii) 
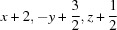
.

**Table 2:** Experimental details

Crystal data	
Chemical formula	C_6_H_10_N_3_O^+^·C_7_H_4_ClO_2_^−^
*M*_r_	295.72
Crystal system, space group	Monoclinic, *P*2_1_/*c*
Temperature (K)	296
*a*, *b*, *c* (Å)	10.1148 (8), 11.2236 (8), 14.579 (1)
β (°)	120.940 (5)
*V* (Å^3^)	1419.57 (19)
*Z*	4
Radiation type	Mo *K*α
μ (mm^−1^)	0.28
Crystal size (mm)	0.35 × 0.30 × 0.20

Data collection	
Diffractometer	Bruker Kappa APEXII CCD
Absorption correction	Multi-scan (*SADABS*; Bruker, 2004 ▸)
*T*_min_, *T*_max_	0.909, 0.946
No. of measured, independent and observed [*I* > 2σ(*I*)] reflections	10962, 3423, 2125
*R*_int_	0.024
(sin θ/λ)_max_ (Å^−1^)	0.669

Refinement	
*R*[*F* ^2^ > 2σ(*F* ^2^)], *wR*(*F* ^2^), *S*	0.047, 0.152, 0.99
No. of reflections	3423
No. of parameters	188
H-atom treatment	H atoms treated by a mixture of independent and constrained refinement
Δρ_max_, Δρ_min_ (e Å^−3^)	0.26, −0.35

Computer programs: *APEX2*, *SAINT* and *XPREP* (Bruker, 2004 ▸), *SIR92* (Altomare *et al.*, 1999 ▸), *SHELXL2017* (Sheldrick, 2015 ▸), *ORTEP-3* (Farrugia, 2012 ▸) and *Mercury* (Macrae *et al.*, 2008 ▸).

## supplementary crystallographic information

### Crystal data

**Table:**

C_6_H_10_N_3_O^+^·C_7_H_4_ClO_2_^−^	*F*(000) = 616
*M**_r_* = 295.72	*D*_x_ = 1.384 Mg m^−^^3^
Monoclinic, *P*2_1_/*c*	Mo *K*α radiation, λ = 0.71073 Å
*a* = 10.1148 (8) Å	Cell parameters from 3319 reflections
*b* = 11.2236 (8) Å	θ = 4.7–53.1°
*c* = 14.579 (1) Å	µ = 0.28 mm^−^^1^
β = 120.940 (5)°	*T* = 296 K
*V* = 1419.57 (19) Å^3^	Block, colorless
*Z* = 4	0.35 × 0.30 × 0.20 mm

### Data collection

**Table:**

Bruker Kappa APEXII CCD diffractometer	2125 reflections with *I* > 2σ(*I*)
Radiation source: fine-focus sealed tube	*R*_int_ = 0.024
ω and φ scan	θ_max_ = 28.4°, θ_min_ = 2.7°
Absorption correction: multi-scan (SADABS; Bruker, 2004)	*h* = −13→12
*T*_min_ = 0.909, *T*_max_ = 0.946	*k* = −14→14
10962 measured reflections	*l* = −15→18
3423 independent reflections	

### Refinement

**Table:**

Refinement on *F*^2^	Secondary atom site location: difference Fourier map
Least-squares matrix: full	Hydrogen site location: mixed
*R*[*F*^2^ > 2σ(*F*^2^)] = 0.047	H atoms treated by a mixture of independent and constrained refinement
*wR*(*F*^2^) = 0.152	*w* = 1/[σ^2^(*F*_o_^2^) + (0.0728*P*)^2^ + 0.324*P*] where *P* = (*F*_o_^2^ + 2*F*_c_^2^)/3
*S* = 0.99	(Δ/σ)_max_ = 0.003
3423 reflections	Δρ_max_ = 0.26 e Å^−^^3^
188 parameters	Δρ_min_ = −0.34 e Å^−^^3^
0 restraints	Extinction correction: SHELXL2017 (Sheldrick, 2015), Fc^*^=kFc[1+0.001xFc^2^λ^3^/sin(2θ)]^-1/4^
Primary atom site location: structure-invariant direct methods	Extinction coefficient: 0.020 (3)

### Special details

**Table:**

Geometry. All esds (except the esd in the dihedral angle between two l.s. planes) are estimated using the full covariance matrix. The cell esds are taken into account individually in the estimation of esds in distances, angles and torsion angles; correlations between esds in cell parameters are only used when they are defined by crystal symmetry. An approximate (isotropic) treatment of cell esds is used for estimating esds involving l.s. planes.
Refinement. Refinement of F^2^ against ALL reflections. The weighted R-factor wR and goodness of fit S are based on F^2^, conventional R-factors R are based on F, with F set to zero for negative F^2^. The threshold expression of F^2^ > 2sigma(F^2^) is used only for calculating R-factors(gt) etc. and is not relevant to the choice of reflections for refinement. R-factors based on F^2^ are statistically about twice as large as those based on F, and R- factors based on ALL data will be even larger.

### Fractional atomic coordinates and isotropic or equivalent isotropic displacement parameters (Å^2^)

**Table:**

	*x*	*y*	*z*	*U*_iso_*/*U*_eq_	
O3	1.08061 (18)	0.73298 (18)	0.57812 (14)	0.0840 (5)	
N1	0.62562 (18)	0.71035 (13)	0.45066 (13)	0.0513 (4)	
N2	0.61625 (18)	0.91121 (14)	0.47011 (15)	0.0649 (5)	
H1N	0.659761	0.979882	0.489408	0.078*	
H2N	0.518251	0.904882	0.442578	0.078*	
N3	0.85256 (18)	0.82711 (15)	0.52563 (13)	0.0555 (4)	
C8	1.1555 (3)	0.8473 (3)	0.6048 (2)	0.0926 (9)	
H8A	1.258389	0.838988	0.618161	0.139*	
H8B	1.159254	0.877851	0.667577	0.139*	
H8C	1.098704	0.901435	0.546305	0.139*	
C9	0.9288 (2)	0.7287 (2)	0.53678 (16)	0.0616 (6)	
C10	0.8601 (3)	0.6168 (2)	0.50699 (19)	0.0703 (6)	
H10	0.919348	0.548952	0.518504	0.084*	
C11	0.7049 (3)	0.60891 (17)	0.46085 (17)	0.0595 (5)	
C12	0.6128 (3)	0.49752 (19)	0.4188 (2)	0.0872 (8)	
H12A	0.531726	0.509732	0.346014	0.131*	
H12B	0.568913	0.477063	0.461457	0.131*	
H12C	0.678410	0.433995	0.421663	0.131*	
C13	0.6995 (2)	0.81598 (16)	0.48261 (15)	0.0489 (4)	
Cl1	−0.42873 (6)	0.70123 (7)	0.18780 (6)	0.0859 (3)	
O1	0.30336 (15)	0.87707 (12)	0.40502 (13)	0.0710 (5)	
O2	0.32766 (15)	0.68727 (12)	0.37489 (12)	0.0648 (4)	
C1	0.2501 (2)	0.77506 (16)	0.37387 (15)	0.0506 (5)	
C2	0.0808 (2)	0.75559 (16)	0.33048 (14)	0.0455 (4)	
C3	0.0178 (2)	0.64290 (17)	0.30515 (16)	0.0532 (5)	
H3	0.081505	0.577740	0.317181	0.064*	
C4	−0.1384 (2)	0.62496 (19)	0.26221 (16)	0.0591 (5)	
H4	−0.179743	0.548535	0.245653	0.071*	
C5	−0.2314 (2)	0.72173 (19)	0.24443 (16)	0.0552 (5)	
C6	−0.1725 (2)	0.83473 (19)	0.26959 (17)	0.0615 (5)	
H6	−0.237007	0.899426	0.257249	0.074*	
C7	−0.0160 (2)	0.85154 (17)	0.31355 (16)	0.0559 (5)	
H7	0.025012	0.927932	0.332014	0.067*	
H1N1	0.508 (3)	0.700 (2)	0.418 (2)	0.089 (8)*	

### Atomic displacement parameters (Å^2^)

**Table:**

	*U*^11^	*U*^22^	*U*^33^	*U*^12^	*U*^13^	*U*^23^
O3	0.0495 (9)	0.1165 (14)	0.0927 (12)	0.0275 (9)	0.0414 (9)	0.0152 (11)
N1	0.0479 (9)	0.0476 (9)	0.0551 (9)	0.0080 (7)	0.0242 (8)	−0.0032 (7)
N2	0.0389 (8)	0.0483 (9)	0.0946 (13)	0.0010 (7)	0.0251 (8)	−0.0125 (8)
N3	0.0411 (9)	0.0722 (11)	0.0533 (10)	0.0081 (7)	0.0243 (7)	−0.0019 (8)
C8	0.0457 (13)	0.140 (3)	0.0899 (19)	0.0033 (14)	0.0337 (13)	0.0031 (17)
C9	0.0507 (12)	0.0858 (15)	0.0564 (13)	0.0194 (10)	0.0333 (10)	0.0100 (11)
C10	0.0728 (15)	0.0697 (14)	0.0839 (16)	0.0340 (12)	0.0514 (13)	0.0182 (12)
C11	0.0738 (14)	0.0508 (11)	0.0653 (13)	0.0173 (9)	0.0439 (11)	0.0081 (9)
C12	0.112 (2)	0.0478 (12)	0.121 (2)	0.0116 (12)	0.0732 (18)	0.0017 (13)
C13	0.0406 (9)	0.0533 (10)	0.0497 (11)	0.0050 (8)	0.0210 (8)	−0.0029 (8)
Cl1	0.0454 (3)	0.1120 (6)	0.0929 (5)	−0.0142 (3)	0.0302 (3)	−0.0069 (4)
O1	0.0424 (7)	0.0458 (8)	0.1021 (12)	−0.0028 (6)	0.0210 (7)	−0.0149 (7)
O2	0.0454 (8)	0.0481 (7)	0.0872 (11)	0.0023 (6)	0.0242 (7)	−0.0098 (7)
C1	0.0404 (9)	0.0449 (10)	0.0527 (11)	0.0003 (7)	0.0141 (8)	−0.0010 (8)
C2	0.0406 (9)	0.0447 (9)	0.0424 (10)	0.0005 (7)	0.0149 (8)	0.0012 (7)
C3	0.0495 (11)	0.0465 (10)	0.0573 (11)	−0.0031 (8)	0.0230 (9)	−0.0053 (8)
C4	0.0549 (12)	0.0578 (12)	0.0623 (13)	−0.0144 (9)	0.0285 (10)	−0.0095 (10)
C5	0.0416 (10)	0.0722 (13)	0.0468 (11)	−0.0074 (9)	0.0191 (8)	−0.0020 (9)
C6	0.0429 (10)	0.0607 (12)	0.0702 (14)	0.0087 (9)	0.0215 (10)	0.0076 (10)
C7	0.0442 (10)	0.0459 (10)	0.0643 (12)	0.0007 (8)	0.0184 (9)	0.0034 (9)

### Geometric parameters (Å, º)

**Table:**

O3—C9	1.331 (2)	C12—H12A	0.9600
O3—C8	1.438 (4)	C12—H12B	0.9600
N1—C13	1.350 (2)	C12—H12C	0.9600
N1—C11	1.356 (2)	Cl1—C5	1.7385 (19)
N1—H1N1	1.03 (3)	O1—C1	1.247 (2)
N2—C13	1.314 (2)	O2—C1	1.255 (2)
N2—H1N	0.8600	C1—C2	1.506 (2)
N2—H2N	0.8600	C2—C3	1.379 (3)
N3—C9	1.308 (3)	C2—C7	1.389 (3)
N3—C13	1.344 (2)	C3—C4	1.383 (3)
C8—H8A	0.9600	C3—H3	0.9300
C8—H8B	0.9600	C4—C5	1.372 (3)
C8—H8C	0.9600	C4—H4	0.9300
C9—C10	1.392 (3)	C5—C6	1.369 (3)
C10—C11	1.356 (3)	C6—C7	1.381 (3)
C10—H10	0.9300	C6—H6	0.9300
C11—C12	1.490 (3)	C7—H7	0.9300
			
C9—O3—C8	118.61 (18)	H12A—C12—H12C	109.5
C13—N1—C11	120.53 (17)	H12B—C12—H12C	109.5
C13—N1—H1N1	124.1 (13)	N2—C13—N3	119.43 (17)
C11—N1—H1N1	115.4 (13)	N2—C13—N1	117.68 (16)
C13—N2—H1N	120.0	N3—C13—N1	122.89 (16)
C13—N2—H2N	120.0	O1—C1—O2	124.51 (17)
H1N—N2—H2N	120.0	O1—C1—C2	118.10 (16)
C9—N3—C13	116.32 (18)	O2—C1—C2	117.39 (16)
O3—C8—H8A	109.5	C3—C2—C7	118.48 (17)
O3—C8—H8B	109.5	C3—C2—C1	121.00 (16)
H8A—C8—H8B	109.5	C7—C2—C1	120.51 (16)
O3—C8—H8C	109.5	C2—C3—C4	121.22 (18)
H8A—C8—H8C	109.5	C2—C3—H3	119.4
H8B—C8—H8C	109.5	C4—C3—H3	119.4
N3—C9—O3	119.7 (2)	C5—C4—C3	118.90 (18)
N3—C9—C10	123.68 (19)	C5—C4—H4	120.5
O3—C9—C10	116.64 (19)	C3—C4—H4	120.5
C11—C10—C9	118.62 (18)	C6—C5—C4	121.40 (18)
C11—C10—H10	120.7	C6—C5—Cl1	119.04 (16)
C9—C10—H10	120.7	C4—C5—Cl1	119.55 (16)
C10—C11—N1	117.9 (2)	C5—C6—C7	119.23 (18)
C10—C11—C12	125.36 (19)	C5—C6—H6	120.4
N1—C11—C12	116.74 (19)	C7—C6—H6	120.4
C11—C12—H12A	109.5	C6—C7—C2	120.74 (18)
C11—C12—H12B	109.5	C6—C7—H7	119.6
H12A—C12—H12B	109.5	C2—C7—H7	119.6
C11—C12—H12C	109.5		
			
O1—C1—C2—C3	−173.20 (19)	C4—C5—C6—C7	0.2 (3)
O1—C1—C2—C7	7.8 (3)	C5—C6—C7—C2	1.2 (3)
O2—C1—C2—C3	7.7 (3)	C13—N1—C11—C10	2.1 (3)
O2—C1—C2—C7	−171.35 (18)	C13—N1—C11—C12	−177.2 (2)
C1—C2—C3—C4	−177.99 (18)	C11—N1—C13—N2	179.63 (19)
C7—C2—C3—C4	1.0 (3)	C11—N1—C13—N3	−0.1 (3)
C1—C2—C7—C6	177.29 (19)	C13—N3—C9—O3	179.53 (18)
C3—C2—C7—C6	−1.8 (3)	C13—N3—C9—C10	−0.1 (3)
C2—C3—C4—C5	0.3 (3)	C9—N3—C13—N1	−0.9 (3)
C8—O3—C9—C10	176.7 (2)	C9—N3—C13—N2	179.37 (19)
C8—O3—C9—N3	−2.9 (3)	O3—C9—C10—C11	−177.6 (2)
C3—C4—C5—C6	−0.9 (3)	N3—C9—C10—C11	2.0 (4)
C3—C4—C5—Cl1	178.62 (16)	C9—C10—C11—N1	−2.9 (3)
Cl1—C5—C6—C7	−179.32 (16)	C9—C10—C11—C12	176.3 (2)

### Hydrogen-bond geometry (Å, º)

**Table:**

*D*—H···*A*	*D*—H	H···*A*	*D*···*A*	*D*—H···*A*
N1—H1*N*1···O2	1.04 (3)	1.60 (3)	2.636 (3)	176 (2)
N2—H1*N*···O1^i^	0.86	2.12	2.846 (2)	142
N2—H2*N*···O1	0.86	1.97	2.824 (3)	169
C8—H8*A*···Cl1^ii^	0.96	2.82	3.770 (3)	171

Symmetry codes: (i) −*x*+1, −*y*+2, −*z*+1; (ii) *x*+2, −*y*+3/2, *z*+1/2.
